# Topical Application of *Derris scandens* Gel for Musculoskeletal Pain Relief and Enhanced Fitness Performance: A Randomized Trial

**DOI:** 10.3390/ph19101509

**Published:** 2026-09-23

**Authors:** Bunleu Sungthong, Peeraya Sriphong, Aranya Thitpraset, Antonious Nagel, Ployvadee Sripadung, Somsak Nualkaew, Wanida Caichompoo, Anake Kijjoa, Catheleeya Mekjaruskul

**Affiliations:** 1 Integrative Pharmaceuticals and Innovation of Pharmaceutical Technology Research Unit, Faculty of Pharmacy, Mahasarakham University, Maha Sarakham 44150, Thailand; bunleu.s@msu.ac.th (B.S.); ployvadee.s@msu.ac.th (P.S.); wanida.c@msu.ac.th (W.C.); 2 Faculty of Pharmacy, Mahasarakham University, Maha Sarakham 44150, Thailand; peeraya.s@msu.ac.th (P.S.); aranyathit@gmail.com (A.T.); tony.nagal@hotmail.com (A.N.); somsak.n@msu.ac.th (S.N.); 3 Pharmaceutical Chemistry and Natural Product Research Unit, Faculty of Pharmacy, Mahasarakham University, Maha Sarakham 44150, Thailand; 4 School of Medicine and Biomedical Sciences Abel Salazar (ICBAS) and CIIMAR, Universidade do Porto, 4050313 Porto, Portugal

**Keywords:** delayed onset muscle soreness, *Derris scandens*, pain, CMJ performance, topical gel, skin tolerability, genistein-7-*O*-[α-rhamnosyl-(1→6)-β-glucopyranoside]

## Abstract

**Background/Objectives:** Delayed onset muscle soreness (DOMS) is a common and recurrent symptom in athletes following intensive or eccentric exercise. This study evaluated the efficacy of a topical gel of *Derris scandens* stem extract on pain and countermovement jump (CMJ) performance in athletes with exercise-induced DOMS and assessed skin irritation in healthy volunteers. **Methods:** Genistein-7-*O*-[α-rhamnosyl-(1→6)-β-glucopyranoside] (GG), a chemical marker of *D. scandens*, was quantified by HPLC. In a randomized, double-blind, active-controlled trial, athletes applied *D. scandens* gel (5%) or diclofenac gel (1%) to the painful site twice daily for 7 days. Pain was recorded daily using a visual analog scale, and CMJ was evaluated on days 1, 2, 4, and 8. Changes from the pre-treatment assessment were compared between groups. **Results:** *D. scandens* gel caused no skin irritation in healthy volunteers. Twenty-five athletes completed the trial (*D. scandens*, n = 13; diclofenac, n = 12). Pain scores decreased and CMJ recovered over the treatment period in both groups, with a significant effect of time but no significant treatment-by-time interaction or treatment effect. Pain reduction from day 2 to day 8 did not differ between groups (MD, 1.75; 95% CI, −1.01 to 4.51; *p* = 0.203), nor did CMJ recovery (MD, 1.96 cm; 95% CI, −2.01 to 5.93; *p* = 0.315). No adverse events were reported. **Conclusions:** Topical *D. scandens* gel was well tolerated, and both treatment groups showed reductions in DOMS-related pain and recovery of CMJ performance over the treatment period. Between-group differences were not statistically significant. Trial registration: Thai Clinical Trials Registry TCTR20180608003.

## 1. Introduction

Delayed onset muscle soreness (DOMS) is a symptom that is usually experienced by elite or recreational athletes. Besides the athletes, DOMS can also occur in strenuous and unaccustomed activities of a non-sporting nature, including activities of daily living. As DOMS causes tenderness, muscle soreness, and muscle damage, which result in alterations to muscle function and joint mechanics, a reduction in fitness performance will follow. DOMS is commonly found at the beginning of the sporting season when athletes start training after a period of reduced activity. The onset of DOMS normally occurs within the first 24 h after cessation of exercise. Moreover, DOMS can also happen in unfamiliar high-force muscular work and an abruptly unusual activity resulting from an elongation of the muscle during simultaneous contraction such as downhill running, resisted cycling, ballistic stretching, isokinetic dynamometry, stepping, and eccentric resistance exercise [1]. Musculoskeletal pain was reported to have higher incidence than other injury and illness in athletes. Pain at the lower extremities of body parts showed the highest percentage of incidence at 30.32% in women’s basketball players [2]. Muscle injuries and joint sprains were found to be 31.8% and 19.8% of all injuries reported in elite female cricketers in Australia, respectively [3]. Muscle strains were found to have the highest incidence at 41.2% of all injuries in professional football players in the UK. Among these muscle strains, thigh was the most common site of the injury (31.7%) [4]. Strained or pulled muscle was found to be the most common injury, i.e., 30.9%, among sport or recreational injuries in Queensland, Australia [5]. Many mechanisms have been reported for DOMS including accumulation of lactic acid, muscle spasm, connective tissue damage, muscle damage, inflammation, and enzyme efflux [1]. Several treatment strategies have been introduced to relieve DOMS and to restore the maximum function of the muscle as quickly as possible. Paracetamol and NSAIDs are commonly used for relieving pain associated with musculoskeletal and sports-related injuries [6]. Nevertheless, intolerance and serious side effects on multiple organ systems such as liver failure and kidney failure due to their systemic activity of paracetamol and NSAIDs have been reported [7]. Gastrointestinal side effects associated with orally administered NSAIDs including dyspepsia and gastric ulceration were found to be as high as 20% [8].

*Derris scandens* (Thao-Wan-Priang in the Thai language) is a large, woody evergreen climbing shrub belonging to the family Fabaceae and is distributed throughout Asia. *D. scandens* is used in Ayurvedic medicine, Traditional Chinese Medicine, and Traditional Thai medicine. In Traditional Thai medicine, stems of *D. scandens* are used in a recipe for pain treatment [9]. Due to its efficacy, dry stem powder and 50% ethanolic extract of *D. scandens* are officially listed as herbal medicines in Thailand National List of Essential Medicines (NLEMs) for musculoskeletal pain treatment. The efficacy of the stems of *D. scandens* for treatment of musculoskeletal pain has already been proven by in vitro and in vivo as well as clinical studies. For in vitro studies, *D. scandens* extract has demonstrated significant anti-inflammatory effects. These include decreasing myeloperoxidase (MPO) release, inhibiting eicosanoid synthesis, and reducing leukotriene B4 generation and cyclooxygenase (COX) enzyme activity [10]. Furthermore, the extract and its chemical constituents have been shown to inhibit nitric oxide (NO) production and suppress the expression of inflammatory genes such as inducible nitric oxide synthase (iNOS), COX-2, interleukin-6 (IL-6), and 5-lipoxygenase (5-LOX) [11]. For in vivo studies, intraperitoneal administration of *D. scandens* extract showed a significant decrease of rat paw edema [12]. Furthermore, oral administration of *D. scandens* extract decreased the number of abdominal constrictions and stretching of hind limb in mice induced by acetic acid in writhing test, suggesting its antinociceptive effect [13]. Moreover, topical application of *D. scandens* extract at a dose 2 mg/ear significantly reduced rat ear edema induced by ethyl phenylpropiolate [14]. In clinical studies, the administration of 800 mg/day and 600 mg/day of 50% ethanolic extract of *D. scandens* alleviated pain in patients who had knee osteoarthritis [15] and low back pain [16], respectively. Moreover, 3 g/day and 1.5 g/day of *D. scandens* dried powder also reduced pain in patients who had knee osteoarthritis [17] and chronic arthralgia from chikungunya [18], respectively. All clinical studies showed no significant differences compared to the standard treatment with NSAIDs. Meta-analysis of the randomized controlled trials of the efficacy of *D. scandens* has been performed. The results showed that oral administrations of *D. scandens* extracts were able to reduce pain score with no significant difference from those of NSAIDs [19]. Investigation of mechanisms of *D. scandens* for musculoskeletal pain revealed that anti-inflammatory effect through iNOS, COX-2, IL-6, 5-LOX [11], COX-1 [10], generation of leukotriene B4 (LTB4), reduction of eicosanoid synthesis via COX and lipoxygenase (LOX) enzymes, and reduction of MPO enzyme release were involved [12].

Chemical study of *D. scandens* stem extract revealed the presence of flavonoids, especially isoflavones. Genistein-7-*O*-[α-rhamnosyl-(1→6)-β-glucopyranoside] (GG), which possesses anti-inflammatory and antioxidant effects, has been reported as a biomarker of *D. scandens* [20]. Moreover, in Thai Herbal Pharmacopoeia, GG and genistein are utilized as chemical markers for *D. scandens* stem extracts [21]. These bioactive constituents therefore provide a relevant basis for extract standardization and investigation of potential anti-inflammatory mechanisms.

Previous evidence suggests that *D. scandens* may have potential as an alternative approach for managing musculoskeletal pain. The utilization of herbal medicine manufactured from medicinal plants cultivated in the local area, particularly in Southeast Asia, can stimulate the local agriculture and boosts the economy for sustainability. However, oral preparations of *D. scandens* have been associated with gastrointestinal adverse effects, including dyspepsia, gastric irritation, nausea, vomiting, flatulence, constipation, loose stools, and mucosal dryness [19]. Therefore, the utilization of *D. scandens* in a topical dosage form may provide a convenient route that could reduce gastrointestinal exposure associated with oral administration. Additionally, topical application of *D. scandens* may allow localized delivery to the affected area. Topical application of *D. scandens* extract was able to reduce ear edema in rats, suggesting an anti-inflammatory effect may be achieved by the topical route [14]. It is well established that inflammation plays an important role in musculoskeletal pain [22]. Although the anti-inflammatory and analgesic properties of *D. scandens* have been investigated in preclinical and oral clinical studies, evidence regarding its topical use in humans remains limited. To the best of our knowledge, no clinical study has been evaluated for a topical formulation of *D. scandens* for DOMS-related musculoskeletal pain and recovery of physical performance in athletes. Therefore, this study aimed to develop and standardize a topical gel of *D. scandens* and to evaluate its skin tolerability and clinical effects on pain and countermovement jump (CMJ) performance in athletes with exercise-induced DOMS and compared its effects with those of the topical diclofenac gel. Molecular docking of identified *D. scandens* constituents with COX-2 was also performed to explore their potential interactions with an inflammation-related molecular target.

## 2. Results

Quality control measures were employed to assess the total flavonoid content and quantification of GG (Figure 1), a specific marker compound in *D. scandens* extract. The total flavonoid content was determined to be 28.61 μg of genistein equivalent/mg of the extract. Utilizing HPLC, GG was identified in the *D. scandens* extract with a retention time of 7.3 min, as depicted in Figure 2. The developed HPLC method for the quantification of GG in the *D. scandens* extract was successfully validated, demonstrating acceptable linearity, precision, and accuracy (Table 1). The method exhibited excellent linearity (R^2^ = 0.999) and high precision with low relative standard deviation (%RSD less than 3.0%). The validation parameters confirmed the reliability and suitability of the method for routine quality control of the extract. In this study, the concentration of GG in the *D. scandens* extract was found to be 31.27 mg/g of extract. Additionally, genistein, lupalbigenin, and scandinone (Figure 1) were detected at retention times of 17.3, 26.9, and 30.1 min, respectively (Figure 2).

The 5% *D. scandens* gel was a homogeneous, yellowish-brown gel, with a mild odor of menthol. The gel showed no phase separation, retained a well-formed consistency, and spread readily and evenly when applied to the skin. The pH of the formulation was 5.26, which lies within the range compatible with normal skin (pH 4–6) [23], and the viscosity was 16,700 cP. After six heating/cooling cycles, the color, odor, homogeneity, and gel consistency were unchanged, and no significant changes in pH or viscosity were observed, indicating that the formulation was physically and chemically stable under the conditions tested.

A flow diagram illustrating the enrollment of healthy volunteers in the skin irritation test is presented in Figure 3. Initially, twenty-three volunteers were assessed for eligibility, and three were subsequently excluded due to logistical constraints. Ultimately, twenty volunteers (10 females and 10 males), with an average age of 21.85 years, were included in the study. All enrolled volunteers completed the study as per protocol. Following the application of *D. scandens* gel and gel base for 24 and 48 h, none of the volunteers exhibited signs of skin irritation. These results were classified as negative skin reactions, indicating no evidence of skin irritation following exposure to the tested formulations.

For the efficacy evaluation, forty athletes were initially assessed for eligibility for this study (Figure 4). However, ten athletes were excluded due to logistical issues (n = 2) or failure to meet the inclusion criteria (n = 8). Subsequently, thirty athletes were recruited and randomized to receive either *D. scandens* gel or diclofenac gel application. However, during the course of the study, two participants in the *D. scandens* gel group and two participants in the diclofenac gel group were lost to follow-up. Additionally, one participant in the diclofenac gel group had a co-intervention, leading to a total of 13 and 12 athletes completing the study for *D. scandens* gel and diclofenac gel treatment, respectively. Baseline characteristics, including sex, age, body weight, height, visual analog scale (VAS) score at Day 2, and CMJ at Day 1, did not exhibit significant differences between athletes who applied *D. scandens* gel and diclofenac gel, as shown in Table 2.

Athletes were instructed to induce DOMS, resulting in leg pain the following day. Pain scores of participants on Days 2–8 were assessed daily, with the results depicted in Figure 5. Pain scores gradually decreased from Day 2 to Day 8 in both treatment groups. Two-way repeated-measures ANOVA with Geisser–Greenhouse correction showed a significant effect of time (*p* < 0.0001) but no significant treatment-by-time interaction (*p* = 0.424) or overall treatment effect (*p* = 0.960). Šídák-adjusted multiple comparisons showed no significant between-group differences at any time point. As shown in Table 3, the mean pain-score change from Day 2 to Day 8 was −5.08 in the *D. scandens* gel group and −6.83 in the diclofenac gel group, with a between-group mean difference (MD) of 1.75 (95% CI, −1.01 to 4.51; *p* = 0.203).

For the CMJ performance test, jump height on Days 1, 2, 4, and 8 is illustrated in Figure 6. CMJ values decreased on Day 2 after DOMS induction compared with the pre-DOMS baseline (Day 1) in both treatment groups, and subsequently increased over the treatment period. Two-way repeated-measures ANOVA of the treatment period (Days 2, 4, and 8) showed a significant effect of time (*p* = 0.0033) but no significant treatment-by-time interaction (*p* = 0.617) or overall treatment effect (*p* = 0.750). Šídák-adjusted multiple comparisons showed no significant between-group differences at Days 2, 4, or 8. As shown in Table 3, the mean CMJ change from Day 2 to Day 8 was 5.46 cm in the *D. scandens* gel group and 3.50 cm in the diclofenac gel group, with a between-group MD of 1.96 cm (95% CI, −2.01 to 5.93; *p* = 0.315). Relative to the pre-DOMS baseline, the mean CMJ change from Day 1 to Day 8 was 0.38 cm in the *D. scandens* gel group and −2.58 cm in the diclofenac gel group, with a between-group MD (between-group mean differences) of 2.97 cm (95% CI, −0.82 to 6.75; *p* = 0.116). Additionally, no adverse events were reported in either the *D. scandens* gel or diclofenac gel groups throughout the study period.

To validate the molecular docking protocol, the co-crystallized ligand was re-docked into the active site of COX-2 (PDB ID: 5KIR). The obtained root mean square deviation (RMSD) between the crystallographic and predicted ligand poses was 0.9008 Å (Figure S1), which is well below the acceptable threshold of 2.0 Å, confirming the reliability of the docking protocol. Diclofenac was selected as the positive control due to its well-established inhibitory activity against COX-2. As illustrated in Figure 7, diclofenac exhibited a binding energy of −8.2 kcal/mol. The ligand formed one conventional hydrogen bond with Ser353, together with π–π T-shaped, π-alkyl, and extensive van der Waals interactions.

The docking poses of the four identified compounds of *D. scandens* are shown in Figure 8. The predicted binding energies were −9.0, −8.4, −7.4, and −5.3 kcal/mol for GG, genistein, scandinone, and lupalbigenin, respectively. GG exhibited the lowest binding energy, suggesting the strongest predicted binding affinity toward COX-2. GG exhibited the most favorable interaction profile, forming five conventional hydrogen bonds involving His90, Tyr355, His351, and Asp347, together with carbon–hydrogen bonding, π-cation, π-sigma, π–π T-shaped interactions, and multiple van der Waals contacts surrounding the active site. In addition, genistein displayed a binding energy comparable to diclofenac, forming carbon–hydrogen bond interactions involving Tyr355 and Ser353, multiple π-alkyl interactions, and several van der Waals contacts. Scandinone interacted with COX-2 through one conventional hydrogen bond with Phe470, together with π-cation, π-anion, and hydrophobic interactions. Lupalbigenin formed three conventional hydrogen bonds involving His90 and Tyr355, together with other non-covalent interactions within the ligand–protein complex.

## 3. Discussion

Flavonoids are commonly found in *D. scandens*, as previously reported [20,24]. Previous studies reported a total flavonoids content of approximately 4.93 μg of quercetin equivalents/mg of extract of *D. scandens* extract [24]. In our current investigation, the total flavonoid content was 28.61 μg of genistein equivalents/mg of extract or 2.86%. Direct comparison between these values should be interpreted with caution because different reference compounds were used for flavonoid quantification. Our finding underscores the importance of standardizing herbal extracts to ensure consistent and reliable product quality. GG was selected as the marker compound for quality control because it is specified, together with genistein, as a chemical marker for *D. scandens* stem extract in the Thai Herbal Pharmacopoeia, which can be readily resolved and quantified by HPLC with diode array detection. Its selection therefore reflects both regulatory recognition and analytical suitability. By quantifying key bioactive markers like GG, we can better understand the relationship between specific compounds and their pharmacological effects. This approach not only enhances the reproducibility of clinical trials but also aids the development of well-characterized and standardized herbal products. The GG content in the *D. scandens* extract, prepared in 50% ethanol and standardized prior to administration to the participant, was 31.27 mg/g of extract. This concentration was higher than the 13.1 mg/g of extract reported in previous studies, also using 50% ethanol [11], and the 14.4 mg/g of extract reported for an aqueous extract [20]. This pronounced discrepancy in bioactive constituent levels could be attributed to variations in extraction parameters, such as processing conditions and solvent efficiency, as well as differences in the geographical origin of the raw materials. Furthermore, environmental and edaphic factors, including soil composition, water availability, and cultivation practices, are well-established variables that can profoundly influence the biosynthesis and accumulation of secondary metabolites in medicinal plants. These findings underscore the rich flavonoid content, particularly GG, in the *D. scandens* extract utilized in our study. In previous clinical trials investigating the pain-alleviating properties of *D. scandens* extracts, the extracts were standardized using GG as a marker, but none of these studies disclosed the specific quantity of this marker present in their formulations. In our study, we conducted simultaneous quantitation of flavonoids and GG contents along with efficacy assessments of the *D. scandens* extract, demonstrating the utilization of a standardized extract. Employing a standardized extract ensures the presence of adequate levels of the herb’s constituents, enhancing the consistency and reliability of the product.

*D. scandens* gel was formulated using HEC 6000 as a gelling agent, despite experimentations with other agents such as carbomer 940 and methylcellulose 4000 being carried out. However, HEC 6000 displayed favorable physical attributes and stability, enduring six rounds of heating and cooling cycles. Furthermore, the pH of the *D. scandens* gel was 5.26, falling within the skin pH range known for its non-irritating properties (pH 4–6) [23]. Conversely, when carbomer 940 was employed as the gelling agent, incompatibility issues between carbomer 940 and *D. scandens* extract emerged. Carbomers are known for their incompatibility with phenol [25], which is a common structural feature of flavonoids, the common constituents of *D. scandens*. This observed incompatibility can be attributed to the presence of flavonoids and other phenolic compounds in *D. scandens* extract. In addition, both *D. scandens* and diclofenac gels contained menthol at 0.2% *w*/*w*, which was included as an odoring agent. This concentration is considerably lower than the 3.5% shown to reduce DOMS-induced symptoms in a previous study [26].

This study represents the first investigation into the topical application of *D. scandens* extract in humans. The dosing calculations followed FDA guidelines [27]. The No Observed Adverse Effect Level (NOAEL) was derived from a previously reported safety dose of 600 mg/kg in rats [28]. Using a body surface area conversion factor of 6.2 and a safety factor of 10, the maximum recommended starting dose for humans was calculated to be 580.62 mg/day (=((600/6.2)/10) × 60 kg)). With the gel applied twice daily over four body areas (calves and thighs) at 0.5 g per area, the specific area dose of *D. scandens* gel was 72.58 mg per area (=580.62/(4 × 2)) or 72.58 mg/0.5 g of gel. This translates to a *D. scandens* content of 14.52 g/100 g of gel or 14.52% *w*/*w*, representing the projected upper concentration based on the NOAEL-derived calculation. Additionally, the human efficacy dose was estimated based on rat ear edema reduction (2 mg/ear) [14], yielding a human equivalent dose of 0.32 mg (=2/6.2) when adjusted for body surface area, according to FDA guidelines [27]. Moreover, by applying a safety factor of 10, the *D. scandens* strength in the gel was estimated at 0.032 mg per area. Given the gel application of 0.5 g per area, the specific area dose of *D. scandens* gel was 0.032 mg/0.5 g of gel, equating to 6.4 mg/100 g or 0.064% *w*/*w*. Thus, the projected concentration range for *D. scandens* gel was between 0.064% and 14.52%. Among the concentrations tested (2%, 5%, and 10% *w*/*w*), the 5% *w*/*w* formulation was selected as the optimal choice due to its favorable appearance and stability under heating/cooling cycles.

Skin irritation or adverse skin reactions are potential concerns with any topically applied products. To ensure the safety of the 5% *D. scandens* extract formulation, a human skin irritation test was conducted prior to the clinical trials. The results demonstrated no signs of skin irritation or adverse skin symptoms, indicating that the 5% *D. scandens* gel was well tolerated under the conditions of this study. This finding is significant as it supports the tolerability of *D. scandens* gel, which is crucial for its potential therapeutic application. The lack of irritation is particularly important for ensuring patient compliance and comfort during treatment. Furthermore, these results align with previous studies that reported the safety of *D. scandens* in various forms [15,28], further supporting the tolerability of *D. scandens*. The comprehensive safety evaluation, including preclinical and clinical phases, underscores the importance of thorough testing in the development of new topical therapies. By confirming the absence of skin irritation under the study conditions, this study provides a foundation for future research on the potential clinical use of *D. scandens* gel. The safety data in this study were obtained from two different populations. The absence of skin irritation supports local tolerability in healthy volunteers under short-term occlusive application, whereas the absence of adverse events reported by athletes during the efficacy trial reflects safety during repeated use over the seven-day treatment period.

Topical drugs are applied directly to the skin over the painful or injured region, allowing the active ingredients to act locally while potentially limiting systemic exposure. DOMS is a common symptom following intensive training or exercise, characterized by musculoskeletal pain and reduced fitness performance. In this study, athletes experienced increased pain one day after intensive exercise, confirming the onset of DOMS before the first application of *D. scandens* gel or diclofenac gel. Participants were then applied topically with either *D. scandens* gel or diclofenac gel, and their pain levels were recorded daily using the VAS. Pain scores decreased progressively from Day 2 to Day 8 in both treatment groups. Repeated-measures analysis demonstrated a significant effect of time but no significant treatment-by-time interaction or overall treatment effect. Moreover, the reduction in pain from Day 2 to Day 8 did not differ significantly between the *D. scandens* gel and diclofenac gel groups (mean difference, 1.75; 95% CI, −1.01 to 4.51; *p* = 0.203). These findings indicate that pain decreased during the treatment period in both groups, without evidence of a differential treatment effect between the two topical treatments. The anti-inflammatory mechanisms of *D. scandens* have been previously documented, indicating a multifaceted approach to inflammation modulation. *D. scandens* extract has been shown to inhibit LTB4 production [20]. LTB4 is a potent pro-inflammatory mediator involved in the recruitment and activation of leukocytes at inflammation sites. By reducing LTB4 levels, *D. scandens* extract can effectively diminish the inflammatory response and associated pain. In addition to these mechanisms, *D. scandens* extract also reduces the synthesis of eicosanoids [20], which are signaling molecules that play crucial roles in inflammation and immunity. The reduction in eicosanoid synthesis can lead to decreased inflammation and pain, contributing to the overall effectiveness of *D. scandens* in managing DOMS. Another important anti-inflammatory action of *D. scandens* is the inhibition of MPO release. MPO is an enzyme found in neutrophils that produces reactive oxygen species, contributing to tissue damage and inflammation [20]. By inhibiting MPO release, *D. scandens* helps protect tissues from oxidative damage and further inflammation. Besides these signaling molecules, free radicals also contribute to inflammation by activating phospholipase A2, leukotrienes, and neutrophils [29]. *D. scandens* possesses significant antioxidant properties [24], which may contribute to its pain-relieving mechanisms. The antioxidant effects of *D. scandens* can mitigate the oxidative stress associated with inflammation. Free radicals exacerbate inflammatory processes by damaging cellular components and activating pro-inflammatory enzymes. By neutralizing these free radicals, the antioxidant compounds in *D. scandens* may reduce the overall inflammatory response, thereby alleviating pain. Moreover, the antioxidant activity of *D. scandens* complements its other pharmacological properties, potentially offering a multifaceted approach to pain management. This holistic mechanism of action underscores the therapeutic potential of *D. scandens* in treating conditions where oxidative stress and inflammation play pivotal roles. Further research should explore the specific antioxidant compounds within *D. scandens* and their interactions with inflammatory pathways. Understanding these interactions could provide deeper insights into how *D. scandens* exerts its effects and help optimize its use in clinical settings. Overall, the combination of leukotriene B4 suppression, eicosanoid synthesis reduction, MPO release inhibition, and antioxidant activity underscores the comprehensive anti-inflammatory potential of *D. scandens*. These mechanisms provide a plausible biological basis for the observed reduction in pain and support further investigation of topical *D. scandens* formulation for DOMS-related pain.

In order to predict the potential molecular mechanisms underlying the anti-inflammatory effects of *D. scandens*, an in silico molecular docking study was performed against COX-2, a key enzyme involved in the inflammatory cascade. The docking simulations revealed varying predicted binding affinities among the identified compounds of *D. scandens*. GG and genistein exhibited docking energies of −9.0 and −8.4 kcal/mol, respectively, which were more favorable than that predicted for diclofenac (−8.2 kcal/mol). These findings suggest that GG and genistein may interact favorably with the COX-2 binding site and provide a structural basis for further investigation of their potential contribution to the anti-inflammatory activity of *D. scandens*. Conversely, scandinone (−7.4 kcal/mol) and lupalbigenin (−5.3 kcal/mol) showed less favorable predicted docking energies. However, since docking scores represent in silico predictions, they do not establish biochemical inhibition or confirm that these compounds mediate the clinical effects observed in this study. Further biochemical and pharmacological studies are needed to effectively determine the contribution of these compounds to the in vitro COX-2 inhibition, in vivo anti-inflammatory activity, and clinical efficacy of topical *D. scandens* gel.

The molecular docking results further provided structural insights into the predicted interactions between the identified phytochemicals of *D. scandens* and COX-2. Diclofenac was used as the positive control and formed a hydrogen bond with Ser353 together with multiple hydrophobic interactions. Among the four phytochemicals, GG exhibited the most favorable predicted docking energy toward COX-2. Its binding pose involved multiple hydrogen bonds, π-interactions, and van der Waals contacts that may contribute to ligand stabilization within the binding site. Furthermore, several interacting residues identified in this study, including Arg120, Tyr355, Ser530, Leu531, Phe518, and His90, correspond to residues previously reported to participate in ligand recognition and stabilization within the COX-2 structure [30]. Therefore, the interaction profile of GG provides structural support for its potential contribution to the anti-inflammatory properties of *D. scandens*, although experimental confirmation of COX-2 inhibition is required.

Although no significant difference in the pain trajectory was observed between the two treatment groups, differences in the skin permeability and metabolism of individual chemical constituents of *D. scandens* may influence their topical pharmacological activity. To the best of our knowledge, the skin permeability of bioactive compounds of *D. scandens* has not been previously investigated. In this study, we predicted the log *K_ow_*, molecular weight (MW), and *K_p_* parameters of GG and genistein using the US-EPA’s DERMWIN^TM^ model in the Epi-Suite^TM^ software (version 4.1). The log *K_ow_* values for GG and genistein were −0.31 and 2.84, respectively, and their MWs were 578.53 and 270.24, respectively. The properties of an ideal compound for good skin penetration include an MW < 500 Da and a log *K_ow_* ranging from 1 to 3. Based on these values, GG may not effectively penetrate the skin, whereas genistein has the potential for skin penetration. This suggestion is consistent with the *K_p_* values provided by the Epi-Suite^TM^ software (version 4.1)., which were 5.54 × 10^−7^ cm/h for GG and 0.00371 cm/h for genistein. These results indicate that GG has limited skin permeability, whereas genistein is more likely to penetrate the skin. It is worth emphasizing that GG served in this study as a marker for extract standardization rather than as a confirmed bioactive constituent responsible for the observed clinical effects. Standardization to GG should therefore be regarded as a measure of batch-to-batch consistency rather than as evidence that GG alone accounts for the therapeutic effect.

However, since GG is a glycoside of genistein, it may be metabolized to genistein in the skin upon topical application, which can eventually penetrate into the skin. Such differences in permeability and metabolism could influence the onset and extent of local pharmacological activity. Additionally, genistein has been reported to have a higher efficacy in inhibiting nitric oxide (NO) production, an important mechanism in anti-inflammatory activity, when compared to GG [11]. It is therefore possible that the metabolic conversion of GG to genistein contributes to the topical anti-inflammatory activity of *D. scandens* extract; however, this mechanism remains hypothetical and requires direct investigation. It is important to emphasize that the present study did not measure the concentrations of the chemical constituents of *D. scandens* in the skin or underlying tissues, and the permeability values reported in this work are solely computational predictions rather than experimental measurements. Therefore, the question whether genistein or other constituents reach concentrations sufficient to produce a local pharmacological effect remains to be determined. Local bioavailability following topical application depends not only on the physicochemical properties of the individual compounds but also on the composition of the vehicle, the integrity and thickness of the skin at the application site, and the amount and frequency of application. The pain relief observed in this study is consistent with local activity of the formulation; however, confirmation would require direct measurement of tissue concentrations, for example, by in vitro permeation testing using human or animal skin or by microdialysis in vivo. Future research should therefore focus on in vitro and in vivo skin permeability tests and the formulation development of topical *D. scandens* products, possibly incorporating skin permeation enhancers or transdermal drug delivery systems to improve their permeability and efficacy. These investigations could support the understanding and optimization of *D. scandens* gel for more effective and faster pain relief.

CMJ, a typical stretch-shortening cycle movement, was utilized to assess fitness performance in this study. Eccentric exercise was followed by a reduction in CMJ values on Day 2 compared with the pre-DOMS assessment on Day 1 in both treatment groups, consistent with an impaired physical performance following DOMS induction. Given the goal of DOMS treatment to alleviate muscle soreness and restore muscle function as quickly as possible, recovery of CMJ performance is important for athletes. Strength reduction after eccentric activity can persist for 10 days before returning to baseline levels [1]. During the treatment period, CMJ performance increased over time in both groups, with a significant effect of time but no significant treatment-by-time interaction or overall treatment effect. From Day 2 to Day 8, CMJ increased by 5.46 cm in the *D. scandens* gel group and 3.50 cm in the diclofenac gel group; however, the between-group difference was not statistically significant (MD, 1.96 cm; 95% CI, −2.01 to 5.93; *p* = 0.315). Previous studies have reported that NSAID treatment may alleviate muscle soreness following eccentric exercise without necessarily producing corresponding improvements in indices of muscle damage [31]. The potential mechanisms that may contribute to recovery of muscle function include the antioxidant [24] and vasodilatory effects of *D. scandens* [32]. Inflammation generates reactive oxygen species (ROS), leading to oxidative stress and tissue damage, which in turn cause muscle soreness and reduced fitness performance. The antioxidant activity of *D. scandens* may mitigate these effects by eliminating ROS, thereby reducing oxidative stress and tissue damage, and ultimately relieving muscle soreness and restoring fitness performance. Additionally, intense training or exercise can lead to anaerobic metabolism and lactic acid accumulation in muscles, causing soreness and performance decline [33]. Vasodilation, the widening of blood vessels, plays a crucial role in improving fitness performance by increasing blood flow and oxygen delivery to working muscles. This enhanced blood flow can help clear metabolic byproducts such as lactic acid, which accumulate during intense exercise and contribute to muscle soreness and fatigue [33]. The vasodilatory effect of *D. scandens* may enhance oxygen delivery to muscles, promoting aerobic metabolism and reducing lactic acid accumulation. Previous studies have reported the inhibition of β-adrenergic receptors, stimulation of NO release from vascular endothelial cells [32], and inhibition of phosphodiesterase 5 [34] by *D. scandens*. These mechanisms collectively contribute to the vasodilatory effects of *D. scandens*. By promoting vasodilation, *D. scandens* may facilitate the removal of these byproducts, thereby reducing muscle soreness and supporting recovery of muscle function. Moreover, improved blood flow from vasodilation can enhance nutrient delivery to muscles, enhancing repair and growth processes essential for recovery and performance. This can lead to more efficient muscle recovery, enabling athletes to regain strength and power more rapidly. Additionally, increased blood flow can improve thermal regulation and reduce inflammation, further contributing to the alleviation of DOMS symptoms. In summary, the antioxidant and vasodilatory properties of *D. scandens* provide plausible mechanisms that may contribute to pain relief and recovery of muscle function. However, the absence of significant between-group differences in CMJ outcomes indicates that these proposed mechanisms and their clinical relevance require confirmation in larger studies.

The clinical relevance of the changes observed in both pain and CMJ performance should also be considered. Published recommendations for the interpretation of pain clinical trials regard a reduction in pain intensity of at least 30% as a moderately important improvement, while a reduction of at least 50% is a substantial improvement from the patient’s perspective [35]. Although these benchmarks were developed for chronic pain, they provide a useful reference for interpreting the extent of pain relief observed in this study. In the present study, mean pain scores decreased from 5.18 to near zero in the *D. scandens* gel group and from 6.82 to near zero in the diclofenac gel group between Day 2 and Day 8, corresponding to reductions well above both thresholds. The pain relief achieved in both groups can therefore be regarded as clinically meaningful for athletes experiencing exercise-induced muscle soreness.

For CMJ performance, no minimal clinically important difference has been established for DOMS. A practical benchmark in this setting is the return of jump performance to the level recorded before the eccentric exercise, since restoration of neuromuscular function determines when an athlete can resume normal training. By Day 8, CMJ in the *D. scandens* gel group had returned to the pre-DOMS value (mean change from Day 1, 0.38 cm), whereas the diclofenac gel group remained below its pre-DOMS value (−2.58 cm). Although the between-group difference did not reach statistical significance (MD, 2.97 cm; 95% CI, −0.82 to 6.75; *p* = 0.116), the observation that jump performance had returned to baseline in one group but not another is of potential practical interest and warrants confirmation in adequately powered trials.

This study has some limitations. The trial was conducted in a single institution among university athletes within a defined age range, which may limit the generalizability of the findings to other populations. Although the sample size was calculated to provide 15 participants per group, dropout during the trial resulted in 13 and 12 participants being analyzed in the *D. scandens* and diclofenac groups, respectively. This reduction in the number of participants analyzed may have limited the statistical coherence to detect clinically meaningful differences between treatments, and the resulting per-protocol analysis may introduce attrition bias. Furthermore, the trial was designed as a superiority comparison and was not powered to test equivalence or non-inferiority; therefore, no equivalence margin was pre-specified. The absence of statistically significant between-group differences therefore reflects a lack of evidence of a difference rather than evidence of equivalence between the two treatments. The same consideration applies to the baseline comparisons. Although baseline characteristics did not differ significantly between the two groups, the present sample size provides limited weight for such comparisons, and non-significant *p*-values should not be taken as evidence of baseline equivalence. The pre-treatment pain score was numerically higher in the diclofenac gel group, which may have allowed greater scope for improvement, and this should be considered when interpreting the between-group comparisons.

The trial was designed as an active-controlled comparison with diclofenac gel rather than as an assessment of the absolute efficacy of *D. scandens* gel. In addition, since both groups received active treatment, but no untreated or placebo control group was included, the observed improvements over time cannot be distinguished completely from the natural recovery of DOMS. The treatment period was limited to one week, and the long-term efficacy and safety of topical *D. scandens* gel remain to be determined. Further multicenter studies with larger sample size, an appropriate control group, and longer follow-up are warranted.

## 4. Materials and Methods

### 4.1. Materials

Genistein was acquired from Biopurify Phytochemicals (Chengdu, China), while GG was generously provided by Dr. Somsak Nualkeaw of the Faculty of Pharmacy, Mahasarakham University, Thailand. Lupalbigenin and scandinone were obtained from Sigma-Aldrich (St. Louis, MO, USA). Diclofenac sodium (USP pharmaceutical grade) was sourced from P.C. Drug Center Co., Ltd., Bangkok, Thailand. Hydroxyethyl cellulose (HEC) 6000 was obtained from Shin-Etsu^®^ (Tokyo, Japan). All chemicals utilized in the formulation process were of cosmeceutical grade and procured from Chemipan Corporation Co., Ltd., Bangkok, Thailand. Other solvents and reagents were of analytical grade.

### 4.2. Preparation of D. scandens Extract

The stems of *D. scandens* were procured from Kaewmungkorn Co., Ltd., Bangkok, Thailand, and authenticated by Dr. Somsak Nualkeaw and Dr. Wanida Caichompoo from the Faculty of Pharmacy, Mahasarakham University, Thailand. A voucher specimen (No. DS2018) was deposited at the Faculty of Pharmacy, Mahasarakham University, Thailand, for future reference. The stems were ground into a fine powder using the herbal grinder (Sunsolid, Samut Prakan, Thailand). Subsequently, 50 g of the *D. scandens* powder were added to 500 mL of 50% aqueous ethanol and refluxed for 2 h [15], after which the flask was allowed to cool to a room temperature, and the suspension was filtered through Whatman^®^ filter paper No. 1. The filtrate was then concentrated using a rotary evaporator (Heidolph^®^, Schwabach, Germany) and further lyophilized using a lyophilizer (Scanvac^®^, Lyngby, Denmark). The yield of the extract was 7.97%.

### 4.3. Determination of Total Flavonoid Content

The total flavonoid content in *D. scandens* extract was determined using a previously described UV-Vis spectrophotometric method [36], with genistein as a marker compound. A stock solution of genistein (150 μg/mL) was prepared by dissolving genistein in methanol. Aliquots of the stock solution were transferred to volumetric flasks containing 1 mL of 2% AlCl_3_ in methanol and adjusted with methanol to obtain final concentrations of genistein ranging from 1 to 30 μg/mL. The *D. scandens* extract was dissolved in methanol to achieve a concentration of 2 mg/mL. Blank solutions for genistein and *D. scandens* extract were prepared following the same procedure but without adding 2% AlCl_3_ solution. Absorbance measurements were performed using a UV-visible spectrophotometer (Jasco V-350^®^, JASCO Corporation, Tokyo, Japan) at 382 nm (n = 3). A standard curve of genistein was constructed by plotting genistein concentrations (*X*-axis) against the absorbance (*Y*-axis). The total flavonoid content in the *D. scandens* extract was determined from the standard curve as mg of genistein equivalent per mg of extract.

### 4.4. High-Performance Liquid Chromatography (HPLC) Analysis

The amount of GG in *D. scandens* extract was determined using the HPLC system (Hitachi Primaide MP-1000 series, Hitachi Ltd., Tokyo, Japan). A C18 column (4.6 × 150 mm, 5 μm particle size, Dr. Maisch GmbH, Ammerbuch-Entringen, Germany) was employed for separation. The mobile phases consisted of acetonitrile (mobile phase A) and 1.0% acetic acid aqueous solution (mobile phase B), and the elution was performed in a gradient manner at a flow rate of 1.0 mL/min. The process started with 15% of the mobile phase A and gradually increased to 30% over 10 min, followed by an increase to 40% over 4 min. Subsequently, a linear gradient was applied to reach 100% of the mobile phase A over 14 min and maintained for 4 min before returning to the initial conditions over 3 min and was then maintained for 5 min. GG and *D. scandens* extract were dissolved in 50% acetonitrile prior to HPLC analysis. Detection was performed using a Diode Array Detector (DAD) (Hitachi Ltd., Tokyo, Japan) set at a 260 nm (n = 3) with an injection volume of 20 μL. Linearity of the method was established over the concentration range of 1.04 μg/mL to 156 μg/mL. The limits of detection (LOD) and quantitation (LOQ) were determined based on the standard deviation (SD) of the response (LOD = 3 × SD and LOQ = 10 × SD). Inter-day precision was evaluated by analyzing low (52 μg/mL) and high (364 μg/mL) concentration samples over three consecutive days. Accuracy was assessed by spiking the *D. scandens* extract with known concentrations of GG (n = 10) and comparing the peak areas of the spiked samples to those of the unspiked extracts.

### 4.5. Preparation of D. scandens Gel and Diclofenac Gel

The gel formulation containing 5% of *D. scandens* extract was prepared employing HEC 6000 as a gelling agent. HEC 6000 (4%) was dispersed in water and heated until gel formation was achieved. Subsequently, a solution of *D. scandens* extract was prepared by dissolving the extract in a mixture of polyethylene glycol (PEG) 400 (5%) and ethanol (5%). Menthol (0.2%) was dissolved separately in ethanol. The solutions of *D. scandens* extract, menthol, and paraben were then incorporated into the HEC 6000 gel and homogenized using a homogenizer at 500 rpm for 30 min. Similarly, a diclofenac gel (1%) was prepared using the same formulation components at the same concentrations and the same preparation procedures as those used for the *D. scandens* gel. The concentration of 1% was selected because it corresponds to the strength of topical diclofenac gel products that are commercially available and widely used for the relief of musculoskeletal pain [37]. The same colorant was incorporated into both formulations so that the two gels were visually indistinguishable. Both gels contained menthol and were therefore comparable in odor.

The physical appearance of the gel, including color, odor, homogeneity, phase separation, and ease of spreading on the skin, was examined visually and by manual application. The pH was measured using a pH meter (Mettler, Toledo^®^, Giessen, Germany), and the viscosity was determined using a Brookfield viscometer (Brookfield^®^, Middleboro, MA, USA). Stability was assessed by heating/cooling cycles, in which the formulation was stored at 45 °C for 12 h followed by 4 °C for 12 h, constituting one cycle, for a total of six cycles. Physical appearance, pH, and viscosity were evaluated before and after cycling.

### 4.6. Human Skin Irritation Test

The study was conducted among healthy volunteers residing in Maha Sarakham Province, Thailand, aged between 18 and 45 years old. The sample size was calculated based on a significance level of 0.05 (Zα/2 = 1.96) and a power of 80% (Zβ = 0.84), with an assumed effect size (d) of 2 and a standard deviation (σ) of 1. The calculation yielded a minimum of 15 participants. To account for an anticipated dropout rate of 30%, the sample size was adjusted to 20 healthy volunteers. The study protocol was approved by the Human Research Ethics Committee, Faculty of Pharmacy, Mahasarakham University, on 26 January 2018, with the Approval Code 016/2560 (Figure S2), and the original approval certificate was issued on 29 January 2018. The study was conducted in accordance with the Declaration of Helsinki. Informed consent was obtained from all participating subjects. The trial was registered with the Thai Clinical Trials Registry (TCTR20180608003) and was published in Mahasarakham University Gazette on 8 June 2018 (Figure S3). Informed consent was obtained from all participating subjects prior to their inclusion in the study. Exclusion criteria included a history of allergy to NSAIDs and/or *D. scandens*, concurrent enrollment in other clinical studies, and loss of follow-up. Participants experiencing allergy symptoms such as skin rash, skin edema, and/or shortness of breath within one hour after application of either the *D. scandens* gel or the gel base (negative control) were eliminated from the study. Prior to gel application, the skin was cleansed with water. Subsequently, *D. scandens* gel and gel base (0.1 g each) were applied to the inner upper right arm within designated 1 × 1 cm areas, spaced 3 cm apart. Both areas were then covered with a waterproof plaster for 30 min. After removing the plaster, skin irritation was assessed using the guidelines established by the International Contact Dermatitis Research Group (ICDRG) [38]. Evaluation criteria included negative reaction, doubtful reaction (faint erythema only), weak positive reaction (erythema, infiltration, possibly papules), strong positive reaction (erythema, infiltration, papules, vesicles), extreme positive reaction (intense erythema, infiltrate, coalescing vesicles), and irritant reaction (various morphologies, e.g., soap effect, bulla, necrosis). Participants who exhibited no skin irritation were subsequently administered the gels at the same 1 × 1 cm areas, using a dosage of 0.1 g, and the areas were then covered with a waterproof plaster for an additional 24 h. Skin irritation was re-evaluated after 48 h.

### 4.7. Efficacy Study of D. scandens Gel in Athletes

#### 4.7.1. Design

A randomized, double-blind, and allocation concealment trial was conducted to compare *D. scandens* gel with diclofenac gel. Participants were randomly assigned to one of the two groups by an authorized person independently: one group received *D. scandens* gel, while another received diclofenac gel. The study was conducted among athletes at Mahasarakham University, located in Maha Sarakham Province, Thailand. The study protocol was approved by the Human Research Ethics Committee, Faculty of Pharmacy, Mahasarakham University on 26 January 2018, with Approval Code 016/2560 (Figure S2), and was conducted in accordance with the Declaration of Helsinki. The trial was registered with the Thai Clinical Trials Registry (TCTR20180608003; date: 4 June 2018). Informed consent was obtained from all participating subjects prior to their inclusion in the study.

#### 4.7.2. Sample Size

The sample size was calculated based on a significance level of 0.05 (Zα/2 = 1.96) and a power of 80% (Zβ = 0.84), with an equal allocation ratio between the two groups (r = 1), an assumed difference in means (μ_1_ − μ_2_) of 1.05, and a standard deviation (σ) of 1. The calculation yielded 15 participants per group, resulting in a total of 30 athletes. Since no previous study was available for reference, the above parameters were assumed.

#### 4.7.3. Subjects

Athletes aged between 18 and 45 years old, residing in Maha Sarakham Province, Thailand, and engaged in sports training for a minimum of 6 weeks were recruited for the study. Exclusion criteria included athletes with a recent history of musculoskeletal pain resulting from training or competition within the preceding 2 months, recent use of opioids and/or NSAIDs within the past 7 days, corticosteroid use within the last 60 days, and a documented allergy to NSAIDs and/or *D. scandens*. Participants were recruited through the distribution of flyers in college campuses and gymnasiums, utilizing advertisement materials to inform potential participants about the study. Participant recruitment and follow-up were conducted between 1 March 2018 and 4 October 2018.

#### 4.7.4. Study Sessions

After receiving the approval of the study protocol from the Human Research Ethics Committee, Faculty of Pharmacy, Mahasarakham University, on 26 January 2018 (Approval Code: 016/2560, Figure S2), the meeting for the study entry criteria was held, and the informed consent was obtained from all participating athletes. Participants were then randomly assigned to receive either *D. scandens* gel or diclofenac gel, which were indistinguishable in color and odor and dispensed in identical containers, for a duration of 7 days (Days 2–8). Randomization was performed using permuted blocks of four, stratified by sex, with the allocation sequence generated from a random number table by a researcher who was not directly involved with the participants and who was responsible for assigning the treatments. Allocation was concealed by dispensing both gels in identical opaque tubes, while the color and the menthol odor of the diclofenac gel were matched to those of the *D. scandens* gel so that the two treatments could not be distinguished by appearance or odor. The participants, the researchers interacting with the subjects, and the outcome assessors were blinded to the assigned treatment group. The assessment parameters included the VAS of pain and CMJ, which were evaluated on Days 2–8 and Days 1, 2, 4, and 8, respectively (Figure 9). The VAS pain rating scale ranged from 0 to 10, with zero representing no pain and 10 indicating the worst pain. Participants were instructed to record their pain scores daily using the VAS in a provided diary. Research coordinators contacted participants on Days 1, 2, 4, and 8 to evaluate CMJ performance. CMJ performance assessment followed the method outlined by Liu et al. [39] with minor modifications. Participants were instructed to stand and raise one hand, measuring the distance from the foot to the fingertip. Subsequently, participants performed a rapid squat jump, aiming to touch a tape attached to a fixed point. Arm swing was controlled by placing the non-measuring hand on the hip throughout the jump. On Day 1, after the baseline CMJ assessment, DOMS was induced by having participants perform step-ups and calf raises. On Day 2, pain intensity and CMJ were assessed before the first application of the assigned treatment. To proceed to the treatment, participants were required to have DOMS-induced pain with a minimum VAS score of 5. Following the Day 2 assessments, the designated gel was applied to the calves and thighs twice daily (morning and evening) for 7 consecutive days (Days 2–8). Gel application was standardized to one teaspoon (approximately 0.5 g) per area, and administration times were recorded in the daily diary. Participants also recorded any concomitant therapies and adverse events during the study period in the diary.

The chronology of ethics approval, trial conduct, and registration are summarized in Table S1.

### 4.8. Molecular Docking Study

In this study, the molecular docking method was used to predict the possible binding interactions between the enzyme and the ligand, with diclofenac serving as the positive control compound. The preparation of the enzyme and ligand was performed using AutoDockTools version 1.5.7 (ADT), and binding energies were calculated using AutoDock Vina version 1.2.0. [40]. The three-dimensional structures (3D) of genistein, lupalbigenin, scandinone, and diclofenac ligands were downloaded from PubChem database (http://pubchem.ncbi.nlm.nih.gov/, accessed on 25 March 2025). The GG ligand structure was created and optimized using quantum mechanical calculations via molecular minimization energy in the Avogadro *1.2.0 package* [41]. The structures of cyclooxygenase-2 (PDB: 5KIR) enzyme were downloaded from RCSB Protein Data Bank [30]. The center coordinates of the grid box for 5KIR were set to X = 21.508, Y = 5.744, and Z = 34.687, with dimensions of 40 × 40 × 40 Å. Water molecules were removed before docking, while polar hydrogen atoms and Kollman charges were added using AutoDockTools. BIOVIA Discovery Studio 2021 (version 21.1) [42] and ChimeraX version 1.10.1 [43] were employed to analyze protein–ligand interactions and generate molecular visualizations.

### 4.9. Statistical Analysis

All results were expressed as mean ± standard deviation (SD), whereas longitudinal data in the figures were presented as means with 95% confidence intervals (CIs). Statistical analyses were performed using GraphPad Prism version 11.0.2 (GraphPad Software, Boston, MA, USA). Statistical analysis of normally distributed baseline continuous characteristics between the *D. scandens* gel and diclofenac gel groups was conducted using independent t-tests, whereas sex distribution was compared using Fisher’s exact test. The efficacy analysis represents a per-protocol analysis, comprising 13 participants in the *D. scandens* gel group and 12 participants in the diclofenac gel group. For the efficacy outcomes, changes in pain score and CMJ were calculated for each participant as the endpoint value minus the corresponding reference value. Pain-score change was calculated from Day 2 (post-DOMS, pre-treatment) to Day 8. CMJ changes were calculated from Day 2 to Day 8 to assess recovery during the treatment period and from Day 1 (pre-DOMS baseline) to Day 8 to assess restoration relative to pre-DOMS performance. Between-group differences in these change scores were analyzed using two-sided Welch’s unpaired t-tests and are presented as mean differences (MDs) with 95% CIs. Changes in pain scores over time (Days 2–8) were analyzed using two-way repeated-measures ANOVA, with treatment group and time as factors and the treatment-by-time interaction included in the model. CMJ during the treatment period (Days 2, 4, and 8) was analyzed using the same approach. Sphericity was not assumed, and the Geisser–Greenhouse correction was applied. When comparisons between treatment groups at individual time points were performed, Šídák’s correction for multiple comparisons was used.

A *p*-value < 0.05 was considered statistically significant. Only participants who completed the study were included in the analysis. Participants who were lost to follow-up or who received a co-intervention were excluded, and no imputation of missing data was performed. Skin permeability (*K_p_*), octanol-water partition coefficient (*K_ow_*), and molecular weight (MW) prediction of the chemical compounds in *D. scandens* was conducted using the US-EPA’s DERMWIN^TM^ model in the Epi-Suite^TM^ software (version 4.1).

## 5. Conclusions

This randomized, double-blind, active-controlled trial carried out in this study showed that topical gel containing 5% *D. scandens* extract was well-tolerated and was associated with reductions in DOMS-related musculoskeletal pain and recovery of CMJ performance over a 1-week treatment period. Pain reduction and CMJ recovery did not differ significantly between the *D. scandens* gel and diclofenac gel groups; however, the relative efficacy of the two treatments remains to be established. Together with the phytochemical standardization of the extract, these findings support the therapeutic potential of standardized topical *D. scandens* preparations and warrant further investigation in larger, adequately powered clinical trials, including experimental studies to confirm the COX-2 interactions predicted by molecular docking.

## Figures and Tables

**Figure 1:**
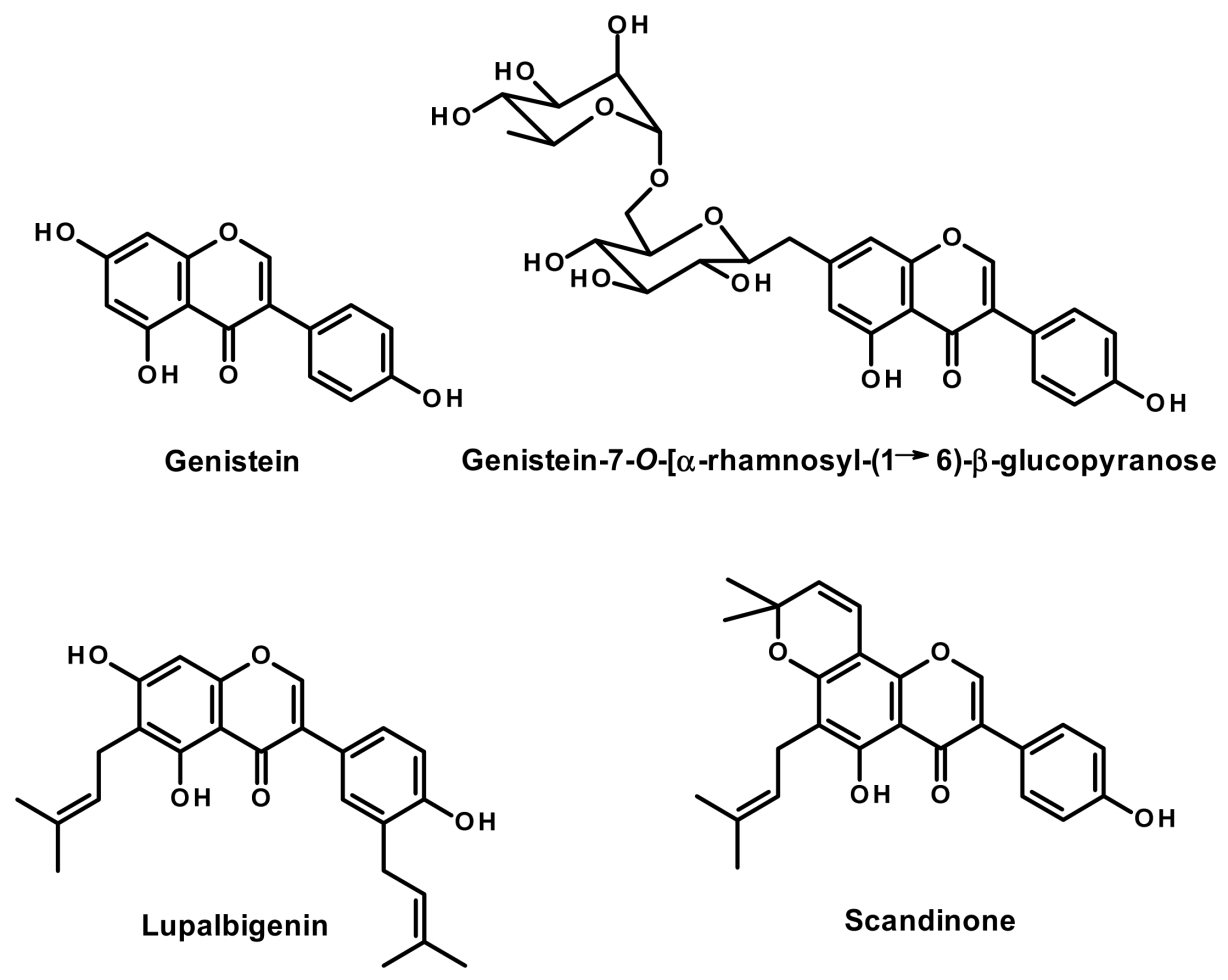
Structures of isoflavone constituent of *Derris scandens*.

**Figure 2:**
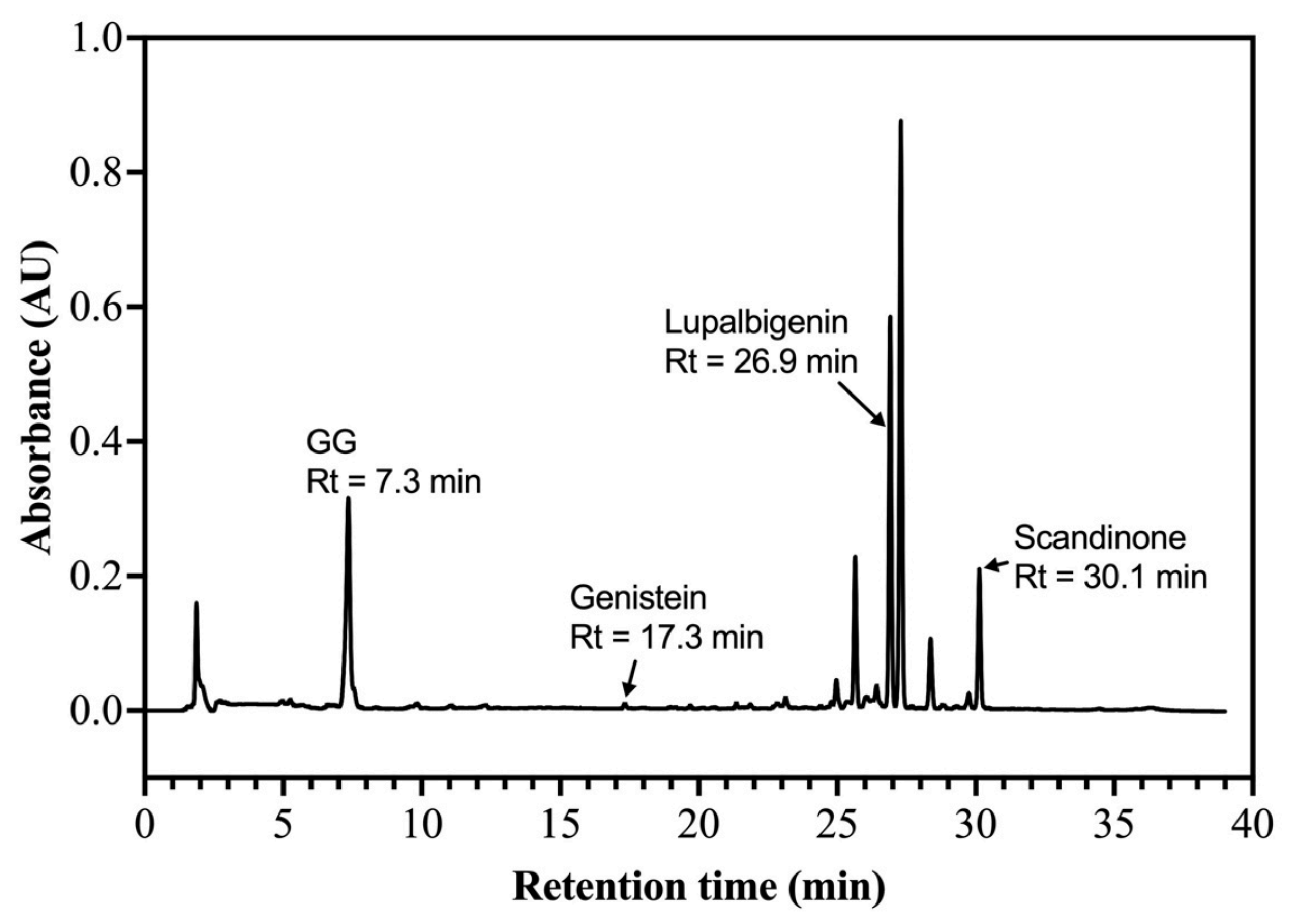
HPLC chromatogram of *D. scandens* extract.

**Figure 3:**
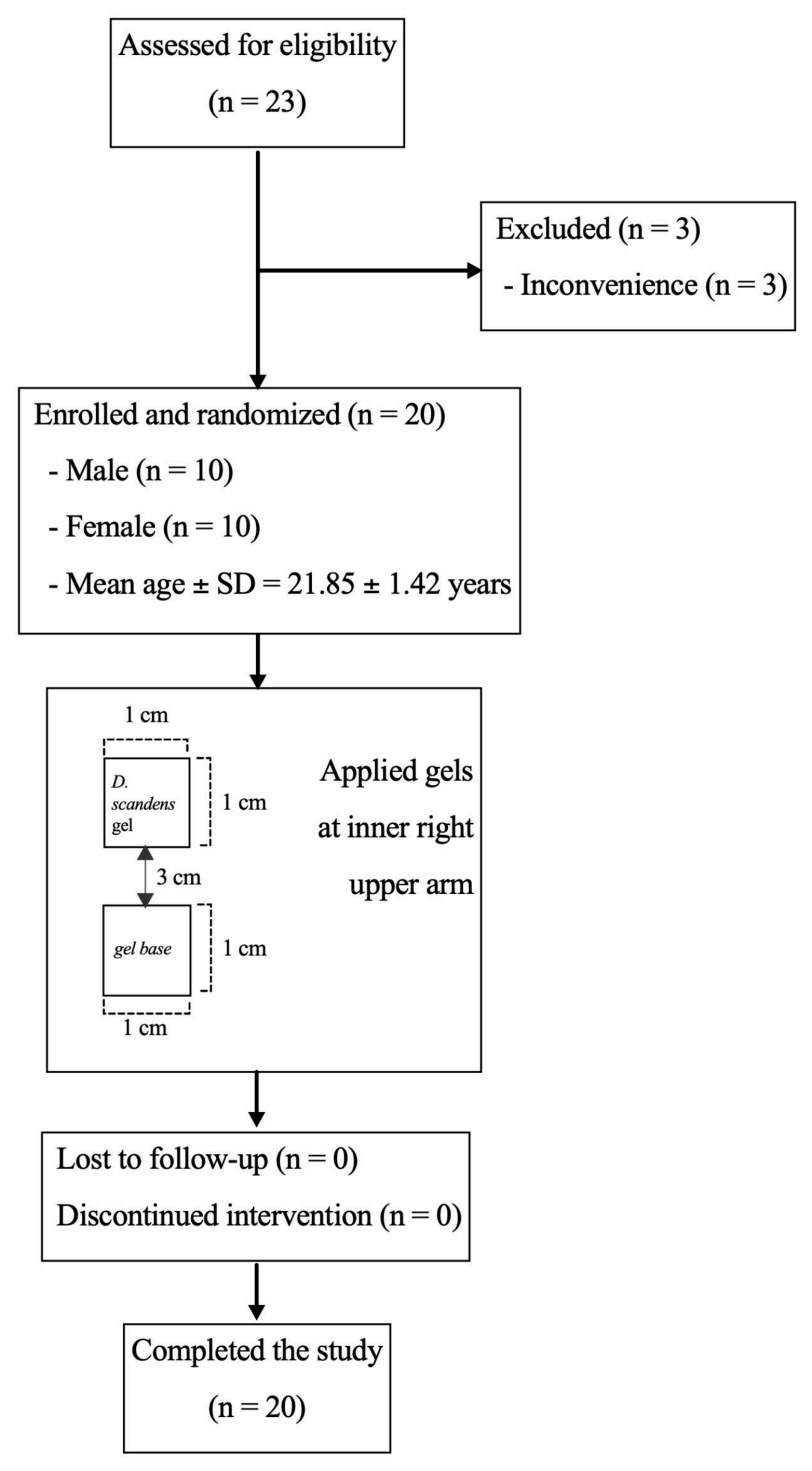
Flow diagram of healthy volunteers in the skin irritation test.

**Figure 4:**
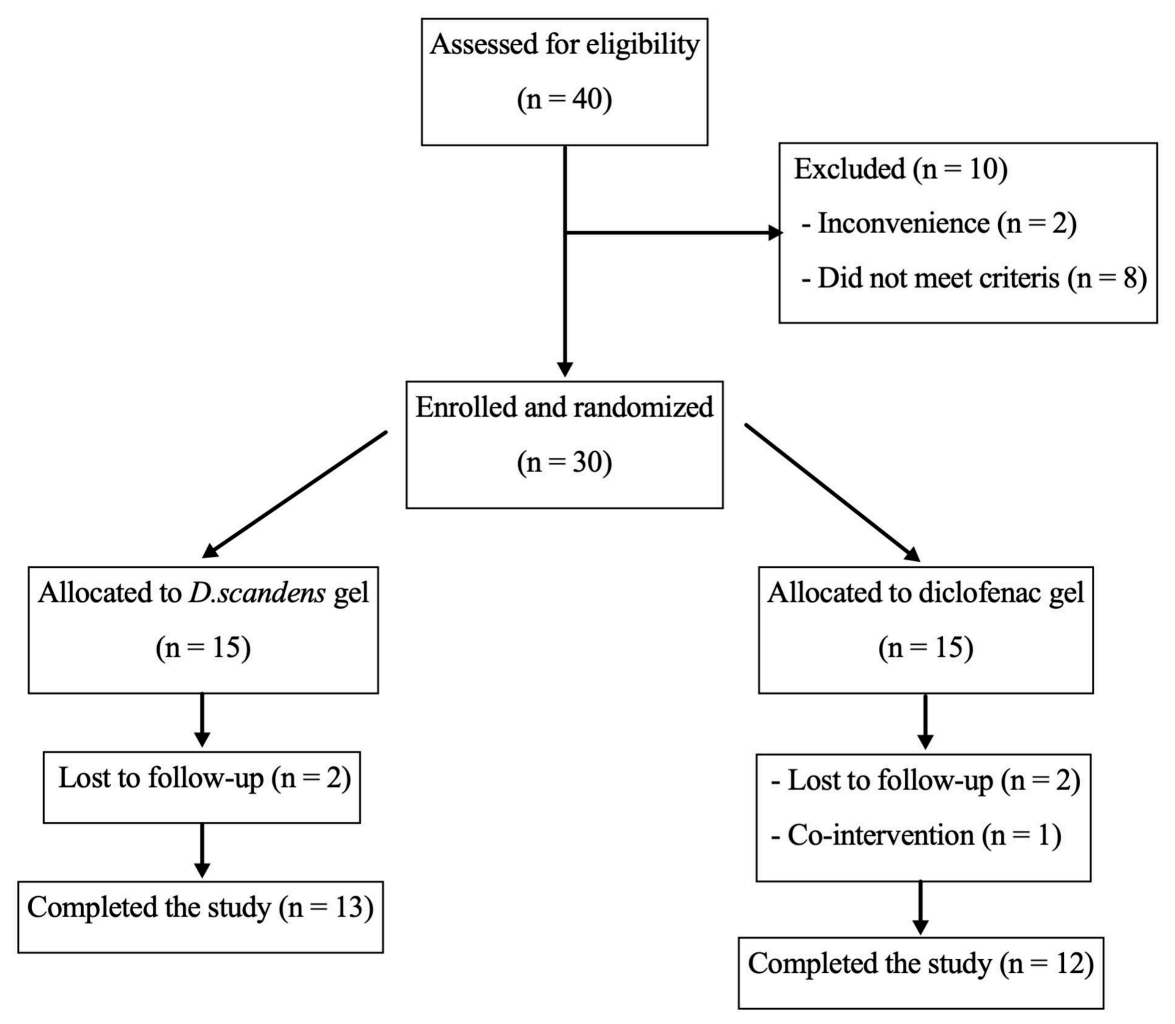
Flow diagram of athletes in the efficacy study.

**Figure 5:**
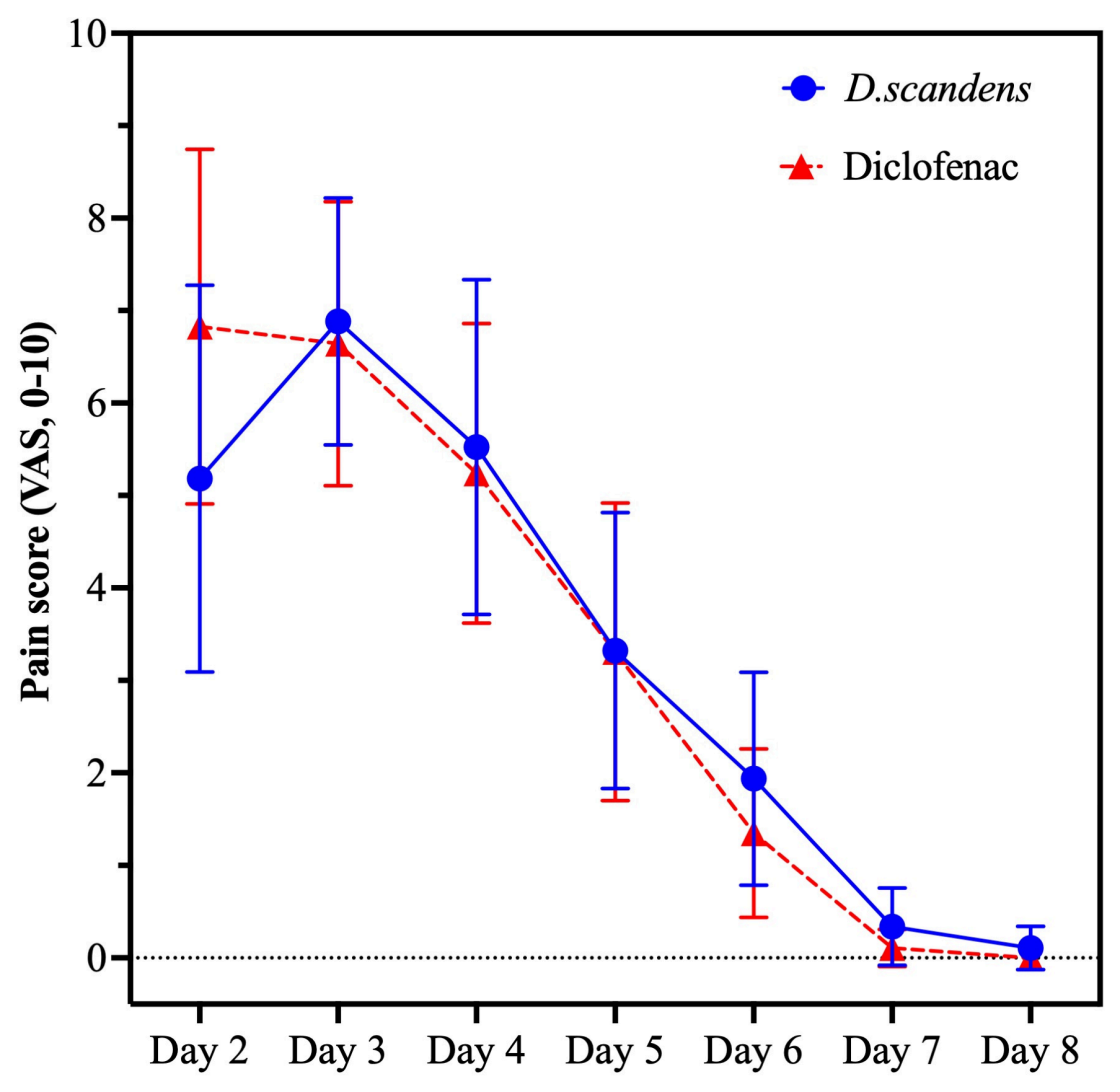
Changes in pain scores over time in the *D. scandens* gel and diclofenac gel groups. Pain scores were assessed from Day 2 (pre-treatment) through Day 8. Data are presented as means with 95% confidence intervals (*D. scandens*, n = 13; diclofenac, n = 12). Two-way repeated-measures ANOVA with Geisser–Greenhouse correction showed a significant effect of time (*p* < 0.0001) but no significant treatment-by-time interaction (*p* = 0.424) or overall treatment effect (*p* = 0.960). Šídák-adjusted multiple comparisons showed no significant between-group differences at any time point.

**Figure 6:**
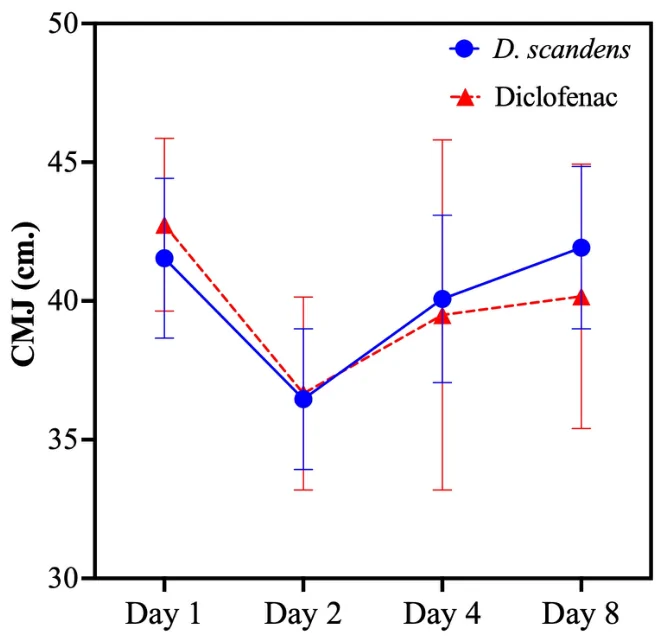
Changes in countermovement jump (CMJ) performance over time in the *D. scandens* and diclofenac groups. Day 1 represents the pre-DOMS baseline, whereas Day 2 represents the post-DOMS, pre-treatment assessment. Data are presented as means with 95% confidence intervals (*D. scandens*, n = 13; diclofenac, n = 12). Two-way repeated-measures ANOVA of the treatment period (Days 2, 4, and 8) showed a significant effect of time (*p* = 0.0033), but no treatment-by-time interaction (*p* = 0.617) or overall treatment effect (*p* = 0.750). No significant between-group differences were observed at individual treatment-period time points after Šídák adjustment.

**Figure 7:**
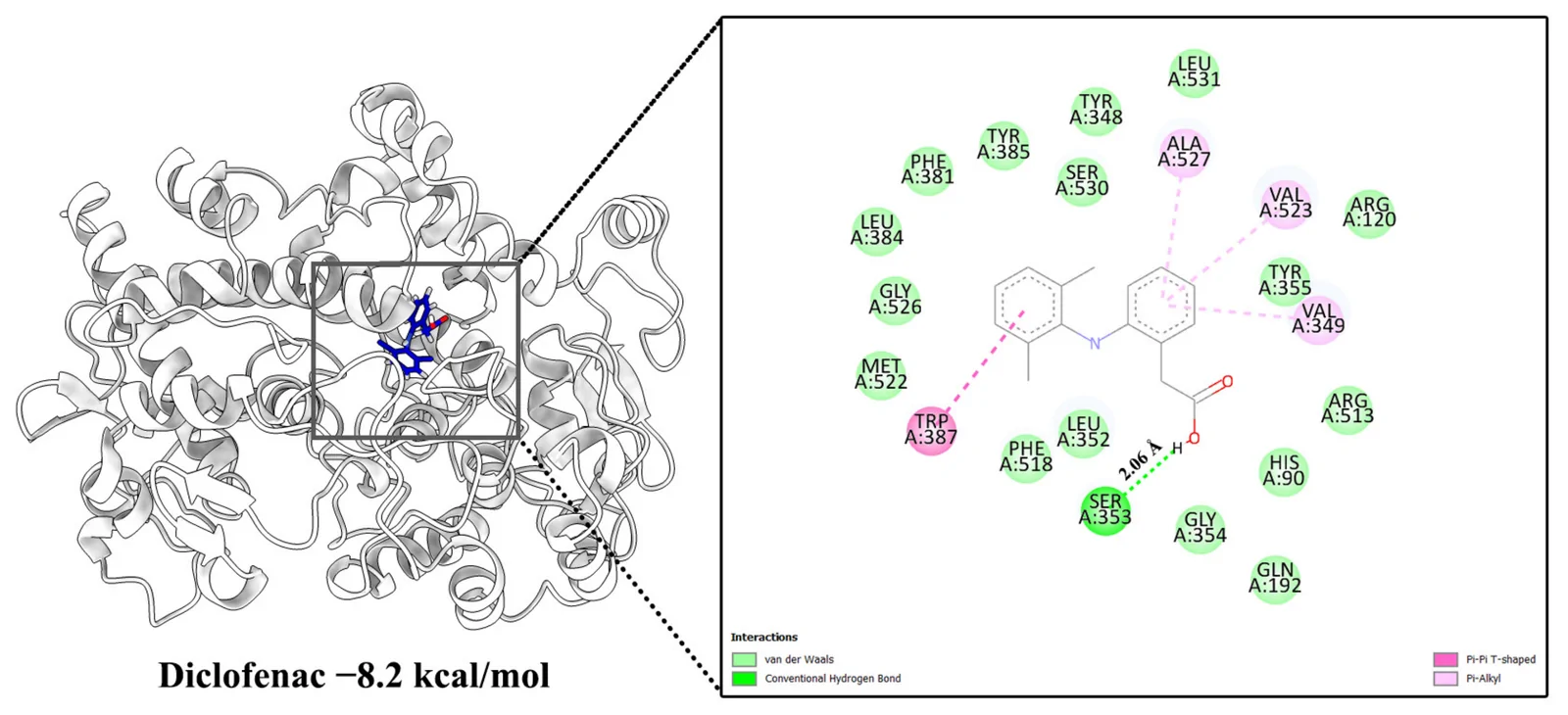
Molecular docking analysis of diclofenac with COX-2.

**Figure 8:**
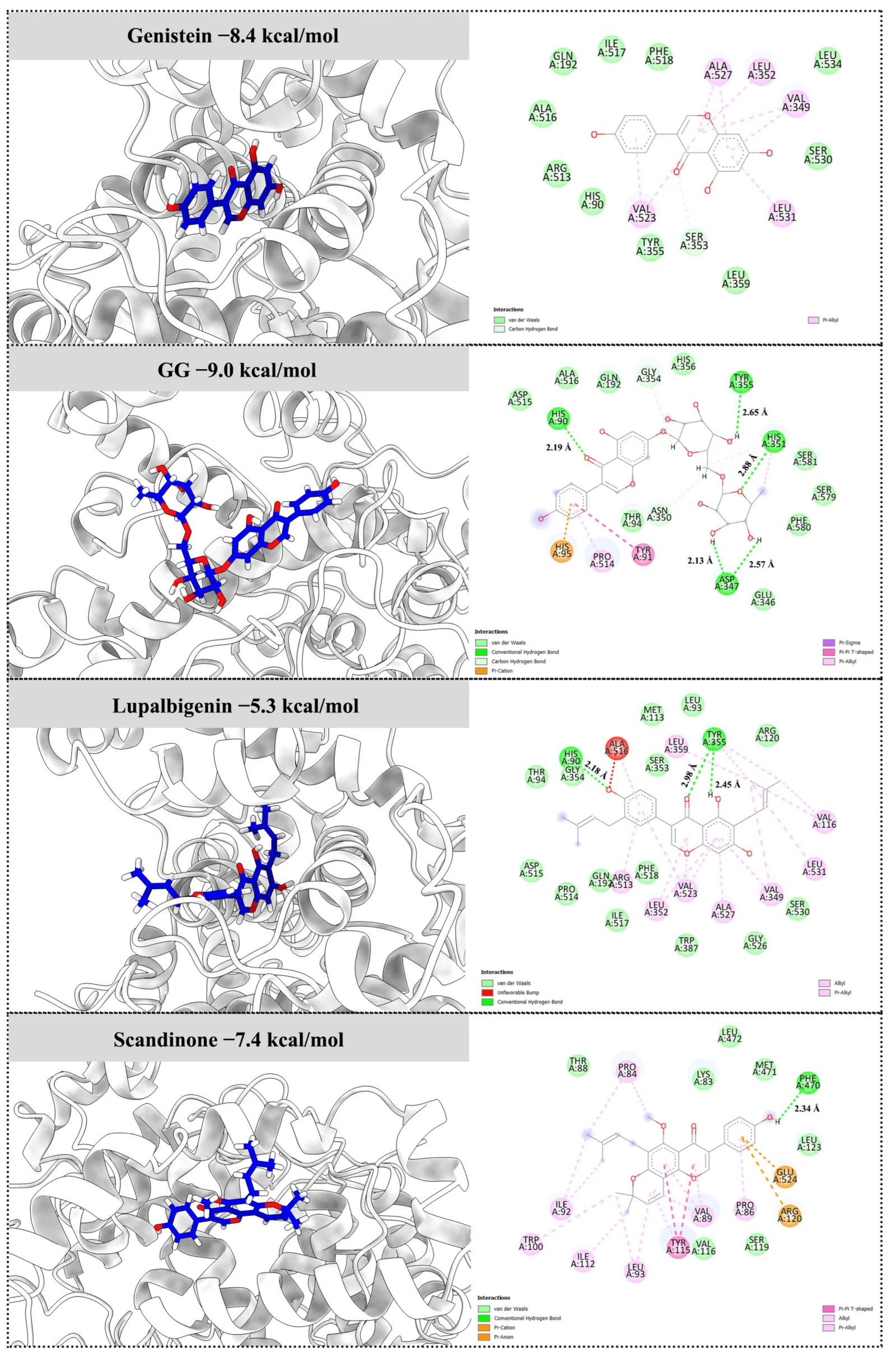
3D and 2D interaction diagrams of genistein, GG, lupalbigenin, and scandinone with COX-2 from the molecular docking analysis.

**Figure 9:**
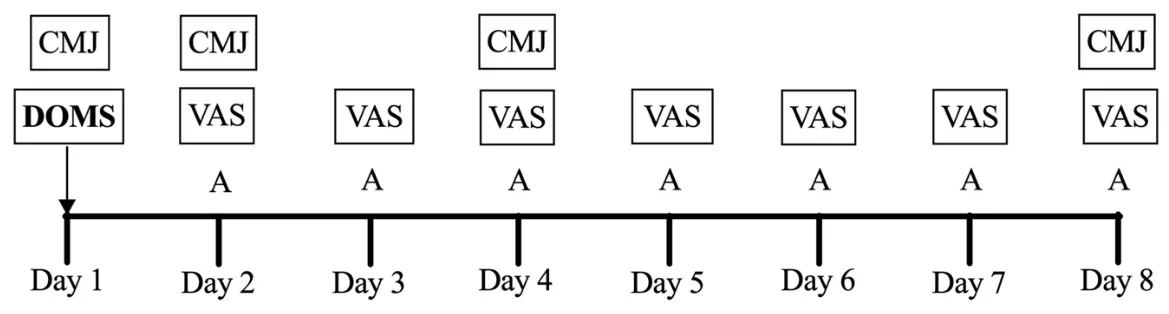
Experimental design for efficacy study in athletes (A: application of *D. scandens* gel or diclofenac gel, CMJ: countermovement jump, DOMS: to do activity for delayed onset muscle soreness, VAS: visual analog scale of pain score).

**Table 1:** Validation parameters of GG in *D. scandens* extract.

Validation Parameters	GG
Precision, %RSD	
Intra-day precision (n = 6)	
Low concentration (52 µg/mL)	2.94
High concentration (364 µg/mL)	2.42
Inter-day precision (3 days, n = 18)	
Low concentration (52 µg/mL)	2.57
High concentration (364 µg/mL)	2.19
%Recovery	
Low concentration (0.51 mg/mL)	105.14
High concentration (1.54 mg/mL)	94.92
LOD (µg/mL)	0.27
LOQ (µg/mL)	0.90
Linearity	
Range (µg/mL)	1.04–156
R^2^	0.999

**Table 2:** Baseline characteristics of 25 athletes.

Characteristics	*D. scandens* Gel (n = 13)	Diclofenac Gel (n = 12)	*p*-Value
Sex: male Mean age ± SD (yr) Mean body weight ± SD (kg) Mean height ± SD (cm) Mean VAS score at day 2 ± SD CMJ at day 1 ± SD (cm)	8 (61.6%) 20.46 ± 1.20 70.92 ± 15.40 172.23 ± 5.45 5.18 ± 3.46 41.54 ± 4.77	8 (66.7%) 19.67 ± 0.65 68.92 ± 11.81 171.75 ± 8.20 6.82 ± 3.02 42.67 ± 9.15	1.000 0.054 0.720 0.863 0.221 0.707

SD: standard deviation, CMJ: countermovement jump, VAS: visual analog scale of pain score.

**Table 3:** Changes in pain intensity and countermovement jump performance.

Outcome	Reference → Endpoint	*D. scandens* (n = 13) Mean Change ± SD	Diclofenac (n = 12) Mean Change ± SD	Between-Group MD (95% CI)	*p*-Value
Pain score	Day 2 → Day 8	−5.08 ± 3.64	−6.83 ± 3.02	1.75 (−1.01 to 4.51)	0.203
CMJ (cm)	Day 2 → Day 8	5.46 ± 3.91	3.50 ± 5.42	1.96 (−2.01 to 5.93)	0.315
CMJ (cm)	Day 1 → Day 8	0.38 ± 3.15	−2.58 ± 5.44	2.97 (−0.82 to 6.75)	0.116

Change was calculated as the endpoint value minus the reference value. Day 2 represented the post-DOMS, pre-treatment assessment for both pain and CMJ, whereas Day 1 represented the pre-DOMS baseline for CMJ. For pain scores, negative change values indicate pain reduction. For CMJ, positive change values indicate improvement in jump performance relative to the corresponding reference assessment. Between-group mean differences were calculated as *D. scandens* minus diclofenac. CI, confidence interval; CMJ, countermovement jump; DOMS, delayed-onset muscle soreness; MD, mean difference; SD, standard deviation.

## Data Availability

The original contributions presented in this study are included in the article/Supplementary Material. Further inquiries can be directed to the corresponding authors.

## Supplementary Materials

The following supporting information can be downloaded at: https://www.mdpi.com/article/10.3390/ph19101509/s1. Figure S1: Redocking validation of the co-crystallized inhibitor into the COX-2 (PDB ID: 5KIR), showing an RMSD value of 0.9008 Å. Figure S2: Ethics approval and certificate granted by the Human Research Ethics Committee, Faculty of Pharmacy, Mahasarakham University (Approval Code 016/2560). Figure S3: Publication of the registration record (TCTR20180608003) in Mahasarakham University Gazette. Table S1: Chronology of ethics approval, trial conduct, and registration.

## Abbreviations

The following abbreviations are used in this manuscript:

**Abbreviation**	**Full term**
GG	Genistein-7-*O*-[α-rhamnosyl-(1→6)-β-glucopyranoside]
CMJ	Countermovement jump
DOMS	Delayed onset muscle soreness
VAS	Visual analog scale of pain score
