# Supplementary material for: Topical Application of Derris scandens Gel for Musculoskeletal Pain Relief and Enhanced Fitness Performance: A Randomized Trial
Source: Pharmaceuticals (Basel). 2026 Sep 23;19(10):1509. doi: 10.3390/ph19101509 (PMC13708325; doi:10.3390/ph19101509)
Supplement: Supplementary file 1 [file pharmaceuticals-19-01509-s001.zip › pharmaceuticals-4560238-supplementary.pdf]

Supplementary Materials

Topical Application of *Derris scandens* Gel for Musculoskeletal Pain Relief and Enhanced Fitness Performance: A Randomized Trial

Bunleu Sungthong [1,2], Peeraya Sriphong [2], Aranya Thitpraset [2], Antonious Nagel [2], Ployvadee Sripadung [1,2], Somsak Nualkaew [2,3], Wanida Chaichompoo[1,2], Anake Kijjoa [4,*] and Catheleeya Mekjaruskul [1,2*]

Figure S1. Redocking validation of the co-crystallized inhibitor into the COX-2 (PDB ID: 5KIR), showing an RMSD value of 0.9008 Å.

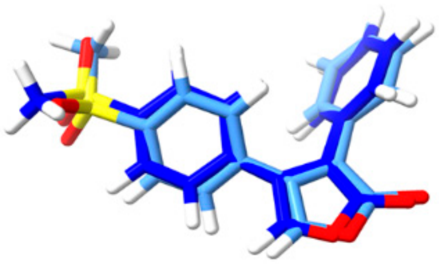

RMSD: 0.9008 Å

Figure S2. Ethics approval and certificate granted by the Human Research Ethics Committee, Faculty of Pharmacy, Mahasarakham University (Approval Code 016/2560)

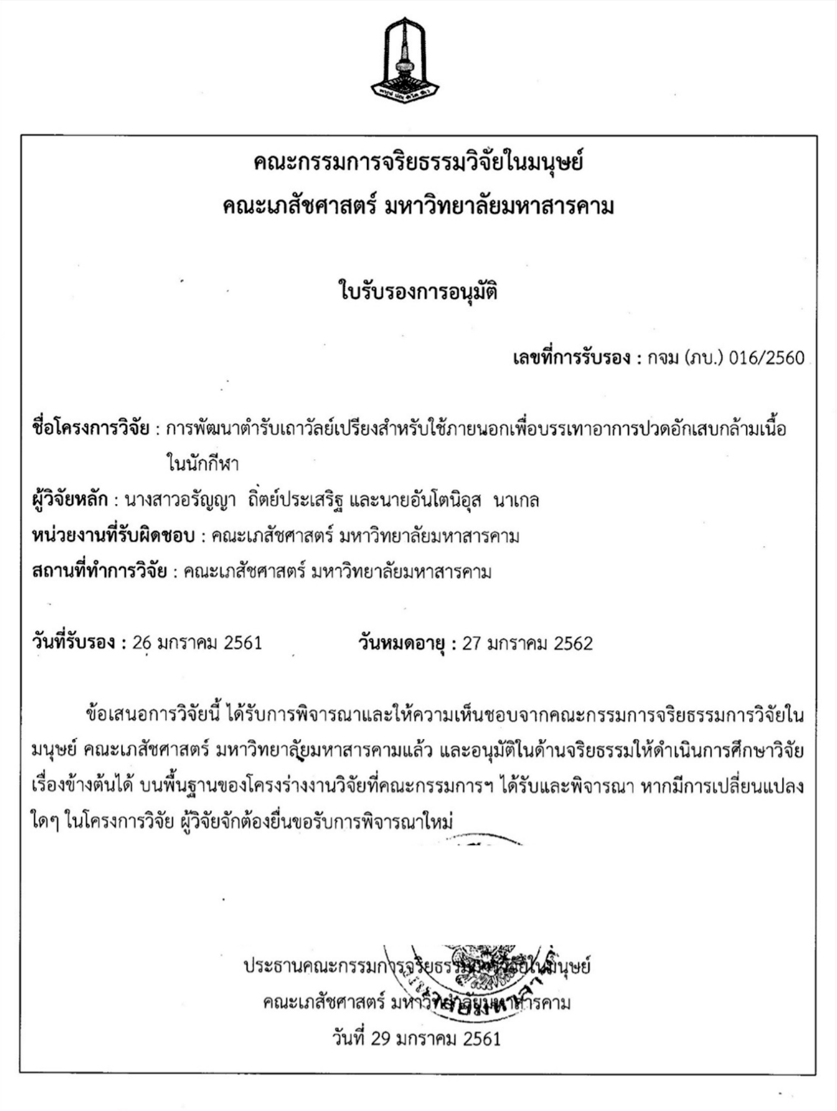

คณะกรรมการจริยธรรมวิจัยในมนุษย์
คณะเภสัชศาสตร์ มหาวิทยาลัยมหาสารคาม

ใบรับรองการอนุมัติ

เลขที่การรับรอง : กจม (ภบ.) 016/2560

ชื่อโครงการวิจัย : การพัฒนาตำรับเถาวัลย์เปรียงสำหรับใช้ภายนอกเพื่อบรรเทาอาการปวดอักเสบกล้ามเนื้อในนักกีฬา
ผู้วิจัยหลัก : นางสาวอรัญญา ถิตย์ประเสริฐ และนายอันโตนิอุส นาเกล
หน่วยงานที่รับผิดชอบ : คณะเภสัชศาสตร์ มหาวิทยาลัยมหาสารคาม
สถานที่ทำการวิจัย : คณะเภสัชศาสตร์ มหาวิทยาลัยมหาสารคาม

วันที่รับรอง : 26 มกราคม 2561 **วันหมดอายุ :** 27 มกราคม 2562

ข้อเสนอการวิจัยนี้ ได้รับการพิจารณาและให้ความเห็นชอบจากคณะกรรมการจริยธรรมการวิจัยในมนุษย์ คณะเภสัชศาสตร์ มหาวิทยาลัยมหาสารคามแล้ว และอนุมัติในด้านจริยธรรมให้ดำเนินการศึกษาวิจัยเรื่องข้างต้นได้ บนพื้นฐานของโครงร่างงานวิจัยที่คณะกรรมการฯ ได้รับและพิจารณา หากมีการเปลี่ยนแปลงใดๆ ในโครงการวิจัย ผู้วิจัยจักต้องยื่นขอรับการพิจารณาใหม่

ประธานคณะกรรมการจริยธรรมการวิจัยในมนุษย์
คณะเภสัชศาสตร์ มหาวิทยาลัยมหาสารคาม
วันที่ 29 มกราคม 2561

Figure S3. Publication of the Registration record (TCTR20180608003) in Mahasarakham University Gazette

Thai Clinical Trials Registry
www.thaiclinicaltrials.org

TCTR ID : TCTR20180608003

OTHER ID :

Overall Recruitment Status : Completed (No Results)

Retrospective registration
This protocol was registered after enrollment of the first participant.

Tracking Information

First Submitted Date :	04 June 2018
First Posted Date :	08 June 2018
Last Update Posted Date :	20 August 2026

Title

Public Title :	Formulation development of Derris scandens gel for muscle pain in athletes
Acronym :	No Data
Scientific Title :	Formulation development of Derris scandens gel for muscle pain in athletes
Sponsor ID/ IRB ID/ EC ID :	N/A
Registration Site :	Thai Clinical Trials Registry
URL :	https://www.thaiclinicaltrials.org/show/TCTR20180608003
Secondary ID :	Other Identifier; Issuing Organization : 000

Ethics Review

1. Board Approval :	Submitted, approved
Approval Number :	016/2560
Date of Approval :	26 January 2018
Board Name :	Ethics committee in human research of Faculty of Pharmacy, Mahasarakham University
Board Affiliation :	Faculty of Pharmacy, Mahasarakham University
Board Contact :	Business Phone : 043754333 Ext. No Data
	Business Email : research.pharm@msu.ac.th
	Business Address : Faculty of Pharmacy, Mahasarakham UniversityKantarawichai District, Maha Sarakham 44150

Sponsor

Source(s) of Monetary or Material Supports :	N/A
Study Primary Sponsor :	N/A
Responsible Party :	Name/Official Title : Faculty of Pharmacy
	Organization : Mahasarakham University
	Phone : 6643754333 Ext. 2707
	Email : catheleeya.m@msu.ac.th
Study Secondary Sponsor :	N/A

Protocol Synopsis

Protocol Synopsis : Delayed onset muscle soreness (DOMS) is common in athletes after intensive or unaccustomed exercise. It causes muscle pain and reduces physical performance. Oral painkillers help but may cause stomach and other side effects. Derris scandens, a Thai medicinal plant listed in the National List of Essential Medicines for musculoskeletal pain, may be a safer option when applied on the skin, but no gel form had been tested in people.
This study evaluated whether a topical gel containing 5 percent D. scandens stem extract relieved muscle pain and restored fitness performance in athletes with DOMS, and whether it caused skin irritation in healthy volunteers.
The study was a randomized, double blind, active controlled trial. Athletes were randomly assigned to apply either D. scandens gel or 1 percent diclofenac gel twice daily for 8 days. Pain was recorded daily in a pain score diary, and jump performance was measured at set time points.

URL not available

Health Conditions

Health Condition(s) or Problem(s) Studied :	Healthy volunteers
Keywords :	Derris scandens, muscle pain, athlete, gel

This document was created on 20 August 2026 at 17:14 (GMT+7).

Metric / Method of measurement : Counter movement jump
Time point : Day 1, 2, 4, 8

Location

Section A : Central Contact

Central Contact First Name : Catheleeya Middle Name : Last Name : Mekjaruskul
Degree : Ph.D Phone : 66817685684 Ext. : No Data Email : catheleeya.m@msu.ac.th

Central Contact Backup First Name : Peeraya Middle Name : Lastname : Sipong
Degree : Ph.D Phone : 66897105987 Ext. : No Data Email : peeraya.s@msu.ac.th

Section B Facility Information and Contact

1. Site Name : Mahasarakham University

City : Kantaraichai State/Province : Maha Sarakham Postal Code : 44150
Country : Thailand Recruitment Status : Completed

Facility Contact First Name : Catheleeya Middle Name : Last Name : Mekjaruskul
Degree : Ph.D Phone : 66817685684 Ext. : No Data Email : catheleeya.m@msu.ac.th

Facility Contact Backup First Name : Peeraya Middle Name : Last Name : Siphong
Degree : Ph.D Phone : 66897105987 Ext. : No Data Email : peeraya.s@msu.ac.th

Investigator Name First Name : Catheleeya Middle Name : Last Name : Mekjaruskul
Degree : Ph.D Role : Principal Investigator

Section C : Contact for Public Queries (Responsible Person)

First Name : Catheleeya Middle Name : Last Name : Mekjaruskul
Degree : Ph.D Phone : 66817685684 Ext. : No Data Email : catheleeya.m@msu.ac.th
Postal Address : Faculty of Pharmacy, Mahasarakham University
State/Province : Maha Sarakham Postal Code : 44150
Country : Thailand Official Role : Study Principal Investigator
Organization Affiliation : Faculty of Pharmacy, Mahasarakham University

Section D : Contact for Scientific Queries (Responsible Person)

First Name : Catheleeya Middle Name : Last Name : Mekjaruskul
Degree : Ph.D Phone : 66817685684 Ext. : No Data Email : catheleeya.m@msu.ac.th
Postal Address : Faculty of Pharmacy, Mahasarakham University
State/Province : Maha Sarakham Postal Code : 44150
Country : Thailand Official Role : Study Principal Investigator
Organization Affiliation : Faculty of Pharmacy, Mahasarakham University

Summary Results

Date of posting of results summaries : Summary results not yet available
Date of first journal publication of results : Not yet published

Deidentified Individual Participant-level Data Sharing

Plan to share IPD : Yes
Plan description : Publishing in journal

Publication from this study

MEDLINE Identifier : No Data
URL link to full text publication : No Data

Eligibility

Inclusion Criteria :	Athlete with eccentric exercise of legs,<br>without the competition for 6 months,<br>live in Maha Sarakham Province,<br>no enroll in other clinical study
Gender :	Both
Age Limit :	Minimum : 18 Years Maximum : 45 Years
Exclusion Criteria :	be injured for 2 months,<br>muscle pain from the exercise before 6 weeks,<br>using the opioids within 7 days,<br>using the corticosteroids both of oral and topical within 60 days,<br>using the non-steroidal anti-inflamatory drugs after injury,<br>using the analgesic drugs after injury,<br>allergy in non-steroidal anti-inflamatory drugs<br>allergy in Derris scandens
Accept Healthy Volunteers :	Yes

Status

Overall Recruitment Status :	Completed	
Key Trial Dates	Study Start Date (First enrollment) : 01 March 2018	Indicate Type : Actual
	Completion Date (Last subject, Last visit) : 04 October 2018	Indicate Type : Actual
	Study Completion Date : 31 December 2018	Indicate Type : Actual

Design

Study Type :	Interventional
Primary Purpose :	Treatment
Study Phase :	Phase 1
Intervention Model :	Parallel
Number of Arms :	2
Masking :	Masked Masked Role : Subject, Investigator, Outcome Assessor,
Allocation :	Randomized
Control :	Active
Study Endpoint Classification :	Safety/Efficacy Study
Sample size	
	Planned sample size : 30
	Actual sample size at study completion : 30
Intervantion Arm 1	
	Intervention name : Derris scandens gel
	Intervention Type : Active Comparator
	Intervention Classification : Drug
	Intervention Description : Derris scandens gel
Intervantion Arm 2	
	Intervention name : diclofenac gel
	Intervention Type : Active Comparator
	Intervention Classification : Drug
	Intervention Description : diclofenac gel

Outcome

Primary Outcome

1. Outcome Name :	Pain level
Metric / Method of measurement :	VAS, Pain score
Time point :	Day 2-8

Secondary Outcome

1. Outcome Name :	Muscle strength

Table S1. Chronology of ethics approval, trial conduct, and registration

Date	Event
26 January 2018	Ethics approval granted by the Human Research Ethics Committee, Faculty of Pharmacy, Mahasarakham University (Approval Code 016/2560). The committee notified the research team by email.
29 January 2018	Date of issue of the signed original approval certificate.
1 March 2018	Enrolment of the first participant.
4 June 2018	Trial record submitted to the Thai Clinical Trials Registry.
8 June 2018	Publication of the Registration record (TCTR20180608003) in Mahasarakham University Gazette.
4 October 2018	Last participant, last visit; data collection completed.
31 December 2018	Study completion.
27 January 2019	Expiry of the ethics approval. All participant-related procedures were completed before this date.
